# INSIGHTS OF HIGHER ORDER MODELLING OF DIFFUSION MAGNETIC RESONANCE IMAGING IN BRAIN INJURY PATIENTS WITH A GLASGOW COMA SCALE OF 13-15: A TRACK-TBI STUDY

**DOI:** 10.51894/001c.122914

**Published:** 2024-08-31

**Authors:** Joshua H. Baker

**Affiliations:** 1 College of Osteopathic Medicine Michigan State University

## 45

### INTRODUCTION

The current nosology of traumatic brain injury has little diagnostic and prognostic value, and the field is moving toward more objective classification of injuries based on validated clinical assessments, neuroimaging, and serum biomarkers. Diffusion tensor imaging is sensitive to the white matter changes following injury but has limited interpretability, especially in voxels containing crossing fibers. Higher-order modeling techniques such as Fixel-based Analysis (FBA) allow more comprehensive questioning of the brain’s white matter with the more flexible signal modeling method constrained spherical deconvolution. In this secondary analysis of diffusion MRI data collected during the TRACK-TBI study, we apply fixed-based analysis to compare white matter differences following injury with controls at two weeks and six months post-injury.

### OBJECTIVE

Examine white matter differences between subjects and controls from the TRACK-TBI study using FBA.

### METHODS

See Figure 1 for a graphic representation of the analysis pipeline.

### RESULTS

Significant white matter regions after family-wise error correction for the FBA metrics Fiber Density (FD), Fiber Cross Section (FC), and Fiber Density Cross Section (FDC) are displayed in Figure 2. FD was lower in mild traumatic brain injury (mTBI) patients than controls in widespread white matter regions, including association fibers, commissural fibers, and projection fibers at both timepoints. At the six-month time point, there was reduced FC in the genu of the corpus callosum.

### DISCUSSION

Our findings reflect a similar pattern to those of a previous NODDI study conducted by TRACK-TBI investigators. The NODDI metric NDI, a metric similar to the FBA metric FD, was lower in mTBI patients compared to controls in a pattern similar to our own study. A critique of the NODDI modeling technique is that it is still a voxel-based method, unlike FBA, which defines a new volume element for comparison, the “fixel.” Ultimately, this study confirms that higher-order modeling techniques are more sensitive to white matter change in those with milder brain injuries; future studies would benefit from collecting data that will allow optimal modeling with these techniques.

